# Supplementary material for: Rapid Screening of Lipase Inhibitors in Scutellaria baicalensis by Using Porcine Pancreatic Lipase Immobilized on Magnetic Core–Shell Metal–Organic Frameworks
Source: Molecules. 2022 May 27;27(11):3475. doi: 10.3390/molecules27113475 (PMC9182405; doi:10.3390/molecules27113475)
Supplement: Supplementary file 1 [file molecules-27-03475-s001.zip › molecules-1729719-supplementary.pdf]

Supporting information

Rapid Screening of Lipase Inhibitors in *Scutellaria Baicalensis* by Using Porcine Pancreatic Lipase Immobilized on Magnetic Core-Shell Metal-Organic Frameworks

Jinfang Xu †, Pengkun Cao †, Zhiyu Fan, Xujing Luo, Gangqiang Yang, Tingli Qu, Jianping Gao*

School of Pharmacy, Shanxi Medical University, 56 Xinjian Road, Taiyuan 030001, People's Republic of China; xujinfang@sxmu.edu.cn (J.X.); caopengkun9257@163.com (P.C.); 13834405334@163.com (Z.F.); Luoxujing08@163.com (X.L.); yanggangqiang2021@163.com (G.Y.); qutingli@126.com (T.Q.)

* Correspondence: Jianping Gao, E-mail: jpgao123@163.com; Tel.: +86-0351-398-5244

† Authors contributed equally to this work

Contents

Figure S1. MS spectrum of the methanol eluent of the ligand fishing by Fe_3O_4-COOH@UiO-66-NH_2@PPL 3

Figure S2. MS/MS spectrum of compound 1 (2′, 3, 5, 6′, 7-pentahydroxyflavanone) 3

Figure S3. MS/MS spectrum of compounds 2 (scutellarein) 4

Figure S4. MS/MS spectrum of compound 3 (baicalin) 4

Figure S5. MS/MS spectrum of compound 4 (oroxyloside) 5

Figure S6. MS/MS spectrum of compound 5 (wogonoside) 5

Figure S7. MS/MS spectrum of compound 6 (skullcapflavone Ⅱ) 6

Figure S8. MS/MS spectrum of compound 7 (wogonin) 6

Figure S9. MS/MS spectrum of compound 8 (oroxylin A) 7

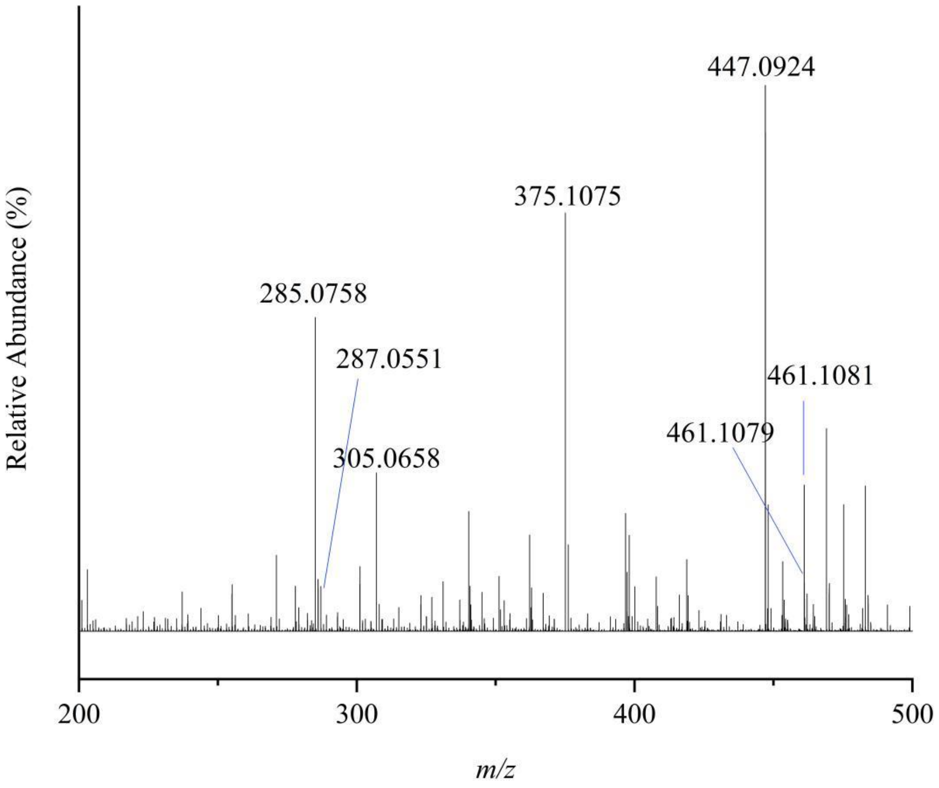

Figure S1. MS spectrum of the methanol eluent of the ligand fishing by Fe_3O_4-COOH@UiO-66-NH_2@PPL

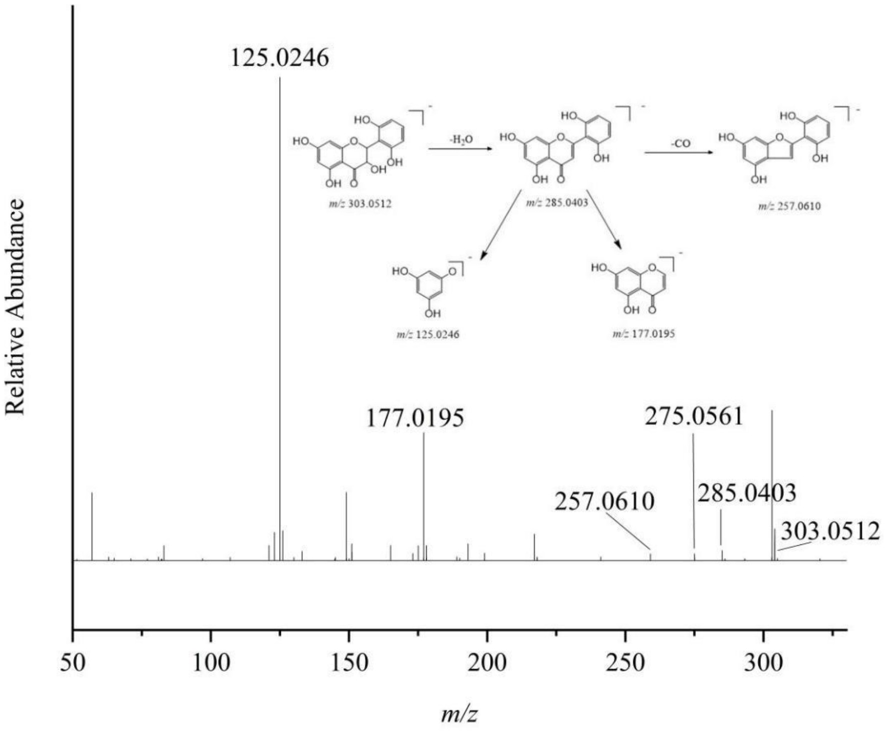

Figure S2. MS/MS spectrum of compound **1** (2′, 3, 5, 6′, 7-pentahydroxyflavanone)

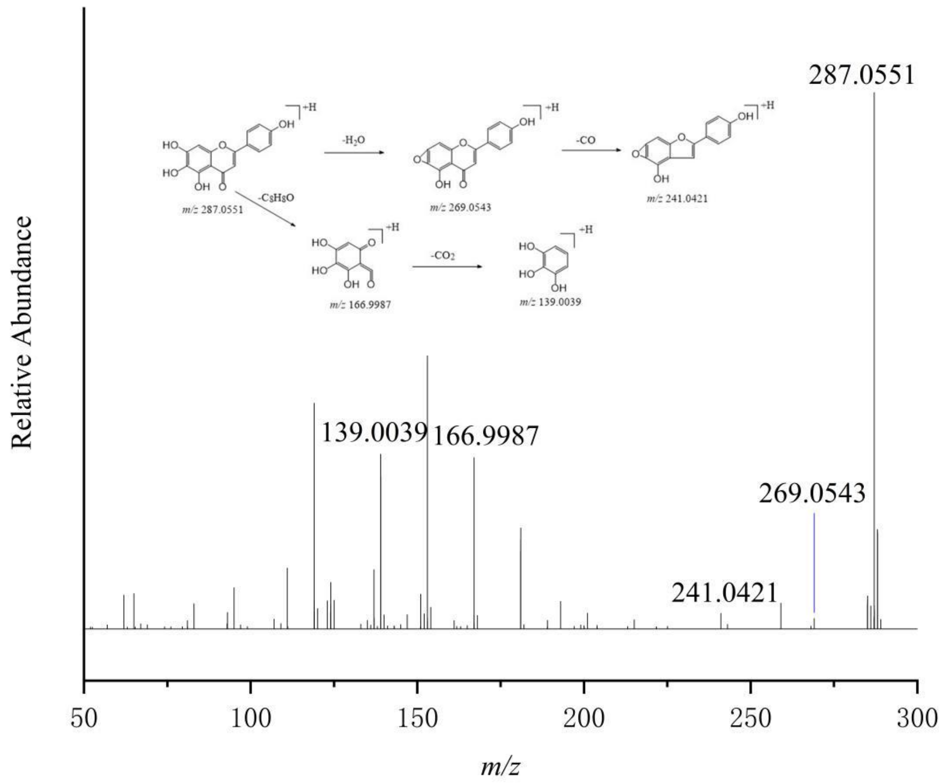

Figure S3. MS/MS spectrum of compound **2** (scutellarein)

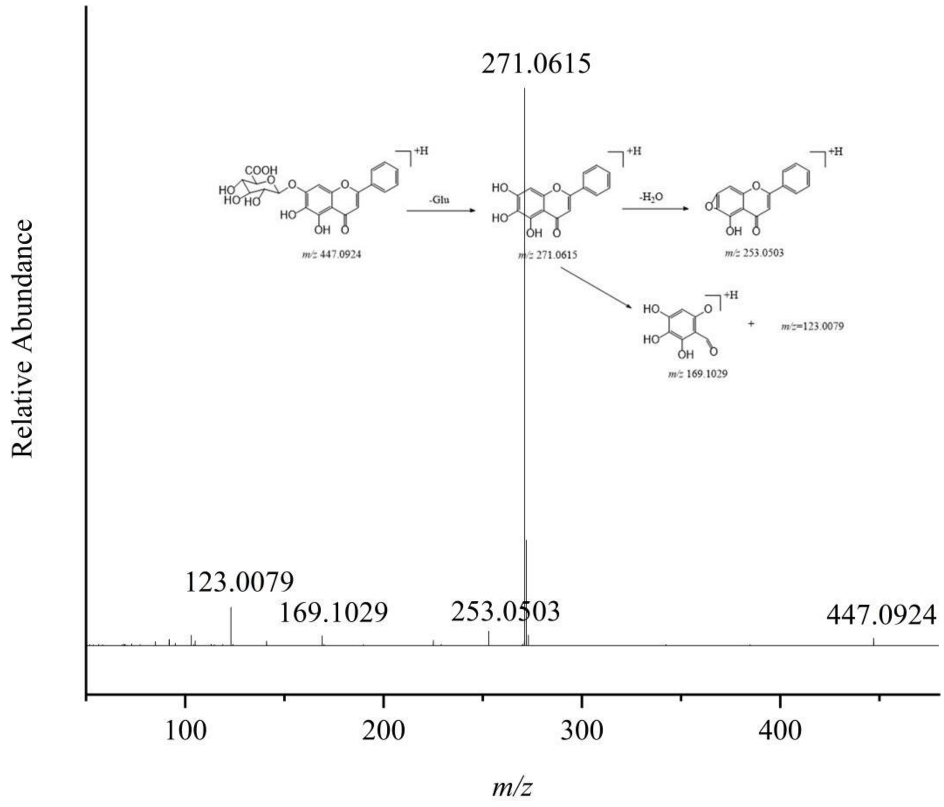

Figure S4. MS/MS spectrum of compound **3** (baicalin)

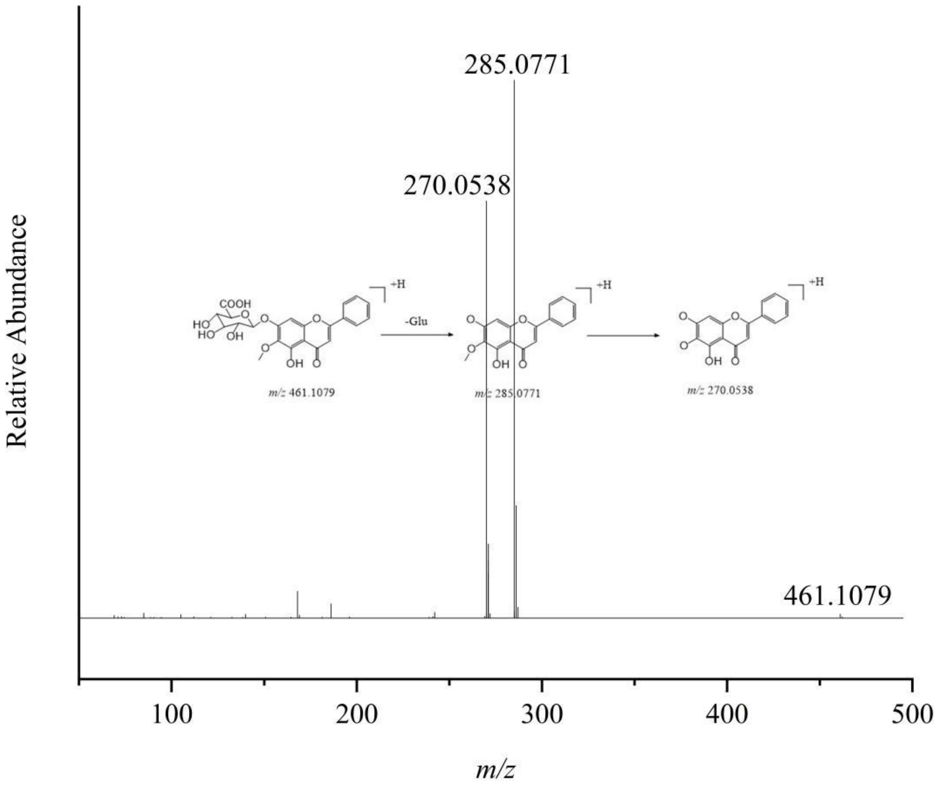

Figure S5. MS/MS spectrum of compound **4** (oroxyloside)

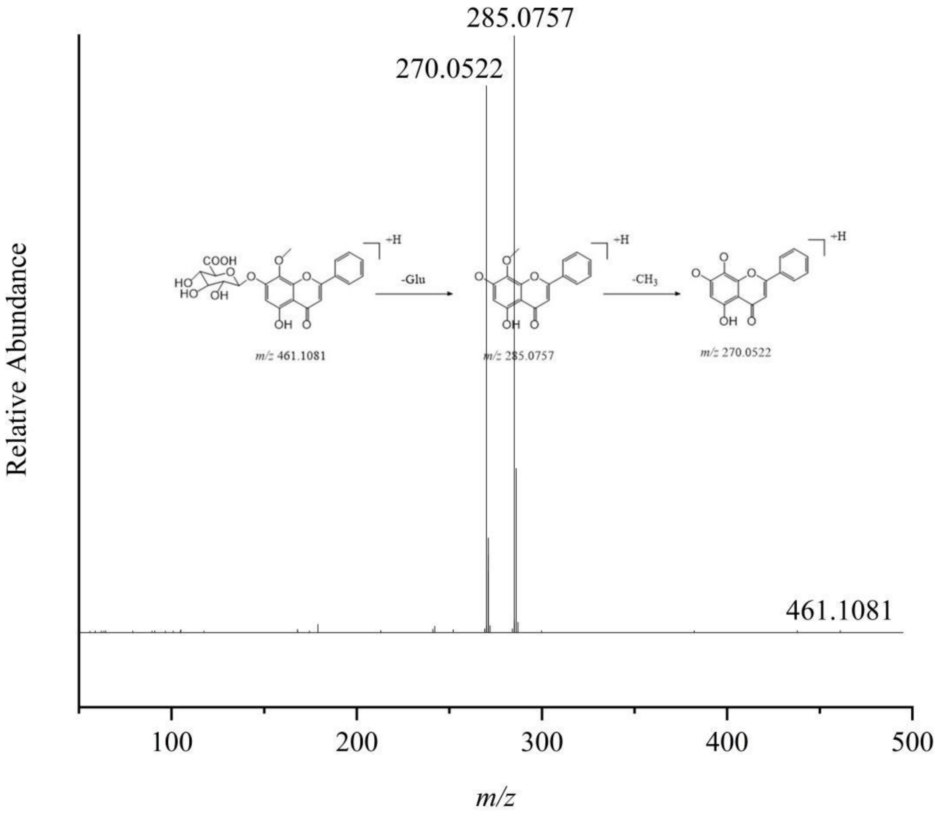

Figure S6. MS/MS spectrum of compound **5** (wogonoside)

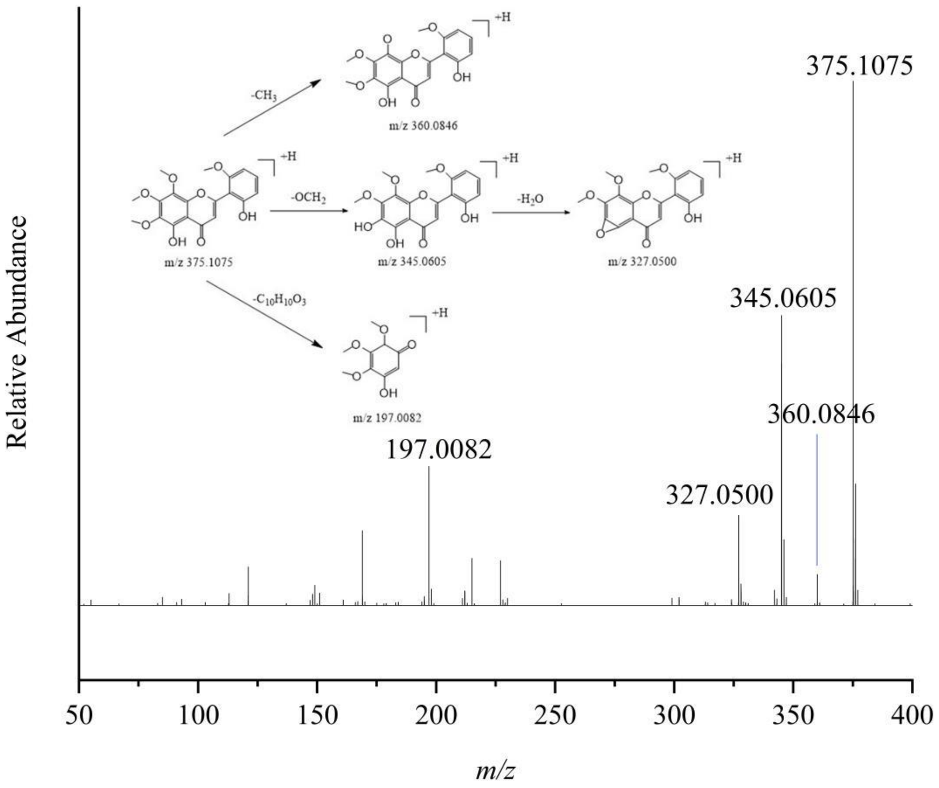

Figure S7. MS/MS spectrum of compound **6** (skullcapflavone II)

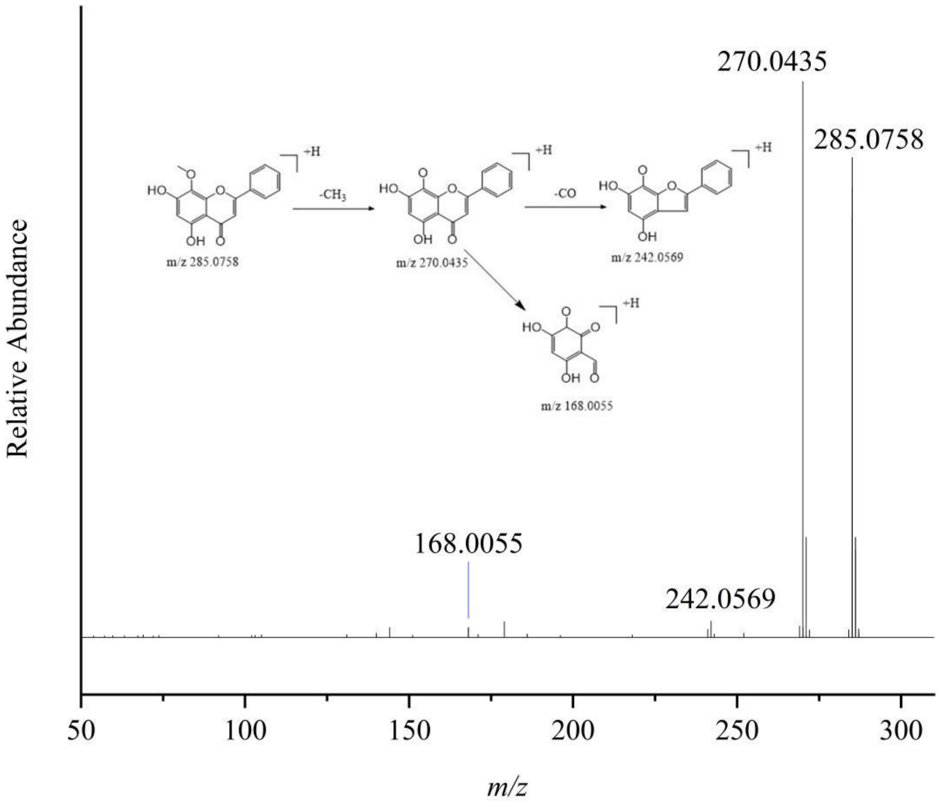

Figure S8. MS/MS spectrum of compound **7** (wogonin)

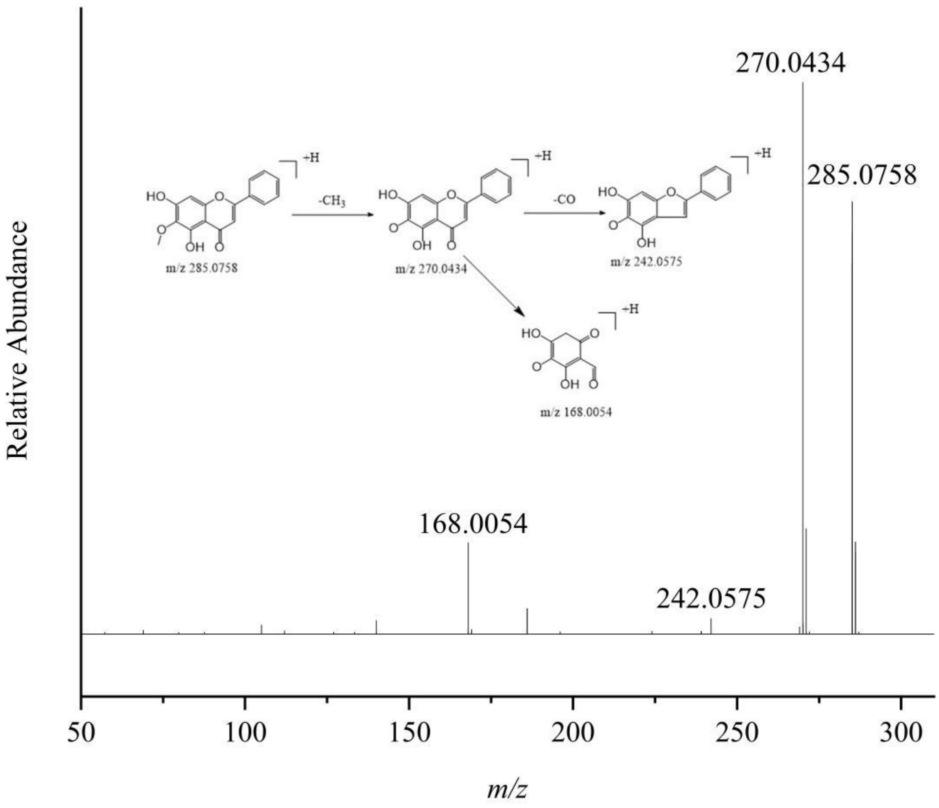

Figure S9. MS/MS spectrum of compound **8** (oroxylin A)
